# The application of traditional transmission electron microscopy for autophagy research in *Caenorhabditis elegans*

**DOI:** 10.1007/s41048-015-0014-z

**Published:** 2015-10-23

**Authors:** Attila L. Kovács

**Affiliations:** Department of Anatomy, Cell and Developmental Biology, Eötvös Loránd University, Budapest, Hungary

**Keywords:** Sample preparation, Fixation, Staining, Dehydration, Embedding

## Abstract

Traditional ultrastructural characterization of autophagic processes remains an important approach to be used in parallel with molecular genetics, light microscopy, and other methods. The special nature of *Caenorhabditis elegans* as an object for transmission electron microscopy makes its introduction into autophagy research a challenging task. The basis of the protocol to prepare *C. elegans* samples for autophagy studies was worked out around the turn of the millennium and has been used since then in my laboratory with some modifications. The method described here enables the user to prepare samples for systematic morphologic as well as morphometric investigations to characterize autophagy with a high but still realistic investment of effort.

## INTRODUCTION

The recent exponential growth of interest in autophagy is taking place in an era of unprecedented progress in methodology of cellular and molecular biology and genetics. While it is the new approaches which are the driving force behind the rapid development of our knowledge, many traditional methods still remain important. Due to the small size of subcellular structures participating in the autophagic process, transmission electron microscopy (TEM) maintains a prominent position in the methodological arsenal for studying autophagy. Innovations involving TEM (e.g., freeze-fracturing, cryofixation, cryosubstitution, immuno-electron microscopy, cryoultramicrotomy, and electron microscopic tomography) have also been and are being developed. They may also be necessary for investigating certain aspects of autophagy; however, their exceedingly high investment needs (in terms of instrumentation, working experience and unit time per sample) make their routine use unrealistic. The traditional TEM approach, however, is at the level of effort which makes it possible to apply it as a tool for regularly and systematically collecting qualitative and quantitative data on subcellular processes (including autophagy) in various cell types.

The sample preparation procedure is one of the key factors for successfully studying cellular structures by TEM. The final result may depend on nuances of the applied technique, which are not written down in sufficient detail in the usual research articles. In addition there are special requirements of sample handling which, to a large extent, are dependent on the properties of the particular type of object we want to study.

*C. elegans* is rather special for studies by TEM in itself (Hall 1995). With no previous results in the literature on autophagy in this important model organism, we took up the challenge and worked out a method of sample preparation in this tiny metazoan for autophagy research. This made us capable of giving the initial qualitative and quantitative description of autophagic structures in various cell types of the “worm” at the subcellular level (Kovács et al. 2004; Sigmond et al. 2008). Here I present the detailed protocol routinely used in my laboratory for preparing *C. elegans* samples to study autophagy by TEM. Some general features, and graphic illustration of certain key steps of the method can be found in a previous article (Sigmond et al. 2008).

Since the first period of its application, the procedure has undergone some changes, as there is always need for improvement in quality, efficiency, and reproducibility. The description below represents the presently used protocol, and I welcome any suggestions and feedback on its application in other laboratories.

## GENERAL CONSIDERATIONS OF SAMPLING

There are two main features of *C. elegans* which basically influence the procedure of sample preparation for TEM. One of them is the rather small size of the body (the diameter and length values are 12–15 and 220–250 µm for freshly hatched larvae and 60–65 and 1000–1300 µm for adults on average, respectively). The other feature is that their cuticule, which covers the whole body, is practically impenetrable to the fixative, which has to diffuse into the cells to carry out its function of properly preserving intracellular structures. The easiest way to overcome the problem of penetration is to cut the worm into pieces in a drop of fixative under a dissection microscope. This action requires higher than average manual skills and makes it necessary, and at the same time possible, to treat the worms individually. Although cumbersome, the need for individual treatment during cutting offers the advantage of allowing observations in single worms which, thereby, can serve as the natural units for both qualitative and quantitative results, and makes it possible to reveal variations among the individuals as well as to carry out proper statistics for morphometry.

*C. elegans* consists of a relatively small number of cells. There are 959 somatic nuclei in an adult hermaphrodite, and consequently considerably less in its various tissues. This allows one to observe characteristic features in the whole worm at the level of individual cells by light microscopy due to the transparency of the body wall. The situation is basically different in TEM studies in which sections must be prepared to reveal the structures inside the cells. Cross sections of the worm’s body are circles which offer only very small areas from each tissue. In addition, as the structure of the body is continuously changing from the tip of the head to the end of the tail, each cross section represents only a certain narrow region. To cover the whole body by cross sectioning would be exceedingly laborious and time consuming for a routine method in autophagy studies. This problem can be overcome by longitudinal sectioning along the antero-posterior axis, which requires proper and exact orientation of the samples. Even in this case, however, we apply serial sectioning. In our method, a set of sections covering five grids satisfy the needs of both qualitative and quantitative observations, giving a good overview of autophagic events at all developmental stages, and in all major cell types of the body (Sigmond et al. 2008).

## FIXATION, INITIAL STAINING, AND WASHING

### Background information

Traditional TEM makes cellular components visible by their selective absorption of heavy metals. Among them, osmium was the first to be employed, based on previous observations in light microscopic histology. Luckily, the solution of osmium tetroxide, osmic acid, can also serve as a fixative for electron microscopy (Palade 1952). Since the introduction of aldehydes [mostly glutaraldehyde, and formaldehyde (Sabatini et al. 1963; Karnovsky 1965)] as primary fixatives, osmic acid has still been kept and used at a later step, called postfixation. Aldehydes and osmic acid are still routinely applied together to give the so called double fixation technique. Other heavy metals, such as uranium and lead, are also used for staining (Glauert 1975). All these heavy metals seem to be absorbed mostly by the same structures, making them non-transparent to the electrons, thereby giving a picture which reflects the metallophilic character of both intra- and extracellular components.

The small size of samples in working with *C. elegans* represents a technical difficulty which seems to be quite formidable at first sight, especially when we have to handle L1 larvae. The cut pieces are so small in this case, that they can be easily mistaken for accidental contaminants. Therefore, one has to rigorously practice cleanliness during sample preparation. Furthermore, containers are needed in which initial staining and washing can be easily performed, and the accidental loss of pieces avoided. To satisfy all these needs we use Terasaki plates (TP) (Sigmond et al. 2008). They have a small volume of about 12–14 µL which, therefore, lose water relatively easily by evaporation. This can be prevented by putting a flat sponge, or layers of appropriately cut filter paper into the plate. Although some of the wells get covered by the wetting layer, the rest are adequate for the work (Sigmond et al. 2008). Even if this wetting method is applied, one has to take care in putting on the cover of the TP whenever it is not actually being used. A daily checking is also advisable when samples are left in the refrigerator in the wells in buffer, waiting for further processing. In the case of visible decrease, the level of the fixative or washing buffer (WB) has to be readjusted with distilled water (DW).

Cutting the worms open for the fixative is carried out under a dissecting stereo microscope with transmitted light illumination at the magnification of ×16 or ×25. In most laboratories, cutting is done with a hypodermic needle which can work well enough. However, we apply broken (from more brittle type) or cut (from softer, flexible type) pieces of razor blades because they are thinner and apply less mechanical pressure on the body during cutting (Sigmond et al. 2008). We use polystyrene Petri-dishes (PD) of microbial quality (bottom or cover) as a cutting surface to give a transparent hydrophobic support of appropriate hardness to preserve the edge of the blade for several rounds of cutting.

### Materials and equipment

- Microbial quality polystyrene PD
- Fixative buffer (FB): it is possible to use a wide variety of recipes, presently we apply 3.2% formaldehyde, 0.2% glutaraldehyde in 0.15 mol/L sodium cacodylate buffer, pH 7.2
- Caution: even at the applied relatively low concentration, aldehydes are irritants and cacodylate is toxic, so avoid contact with skin, especially take care of eyes, and work in a well ventilated room.
- Washing buffer (WB): 0.125 mol/L sodium cacodylate buffer, pH 7.2
- 0.1% ruthenium red solution in DW
- Dissecting stereo microscope
- Inverted microscope with digital camera
- TP with wetted insert of artificial sponge or stack of filter paper
- Wetting solution: 1.5% glutaraldehyde in DW to prevent fungal and bacterial growth
- Adjustable pipettes
- Obliquely cut fine plastic pipette tip (PT) with good transparency
- Hair device: a piece of hair inserted and glued into the tip of a Pasteur-pipette or fine plastic PT; (recently we have started to use the hair of eyelash or eyebrow, because they have a finer tip)

### Procedure

1. Pipette out 100 µL fixative into a PD. On an appropriately hydrophobic surface this volume can spread out as a 10 mm diameter convex drop.
2. Put a single worm into the drop of FB. In fixatives with high formaldehyde content the worms stop wriggling within 10–20 s, while in solutions with glutaraldehyde only, they may keep moving for many minutes.
3. If necessary a photograph of the intact worm in the fixative can be easily made at this stage using an inverted microscope. This may facilitate later orientation inside the TEM sections and the identification of corresponding structures at light microscopy and TEM levels.
4. Cut the worm into maximum 3–4 pieces.
5. Collect the pieces in one group in the center of the drop with the help of the hair device.
6. Using the obliquely cut fine plastic PT, pipette up all pieces with one draw if you can, and release them into the appropriate well of the TP. Check if all pieces are transferred. Repeat the process with pieces left behind.
7. If a piece (or more) is missing it is most likely attached to the wall of the PT. If the PT is transparent enough you can check that in the stereo microscope. This is usually not necessary though, as you can go back to the big drop of FB where you made the cutting, and release the attached piece by vigorously pipetting up and down. In rare cases when a piece attaches to the inside of the PT so tightly that it cannot be detached by pipetting up and down, you can release it under the microscope with the help of the hair device.
8. Always keep track of all the pieces to avoid mixing of samples. If a piece happens to be lost in spite of all efforts, change both the PD with the FB and the used PT to new ones.
9. Fill the well of the TP with fixative to make the surface of the solution slightly convex to act as a lens that makes it easier to recognize the samples.
10. At this stage, we can collect the samples into the center of the well and make photographs again of them. This may be very useful to help in their identification, as the danger of losing a piece is always present in several of the later steps of sample preparation, especially during washing and embedding into agar.
11. Add a few drops (1–2 µL) of 0.1% ruthenium red solution to the fixative until the buffer becomes deep red with the samples remaining clearly visible (ruthenium staining can also be done later in the first washing step). In our experience ruthenium red has the advantage of making the sample pieces better recognizable during the whole preparation procedure.
12. Let the samples stand in the fixative for a few (at least two) hours, or overnight in a refrigerator.
13. Wash the samples with WB. This is done by replacing FB with WB, pipetting down as much of the FB as possible without removing any of the sample pieces. To make washing more efficient, collect samples with the hair device to one side of the bottom of the well, and use a plastic PT with a narrow capillary ending. In one washing round make three exchanges of WB and, altogether, carry out at least three rounds with at least some hours in between. In cacodylate-based WB, fixed samples can be left in the refrigerator for weeks without apparent change. Cacodylate prevents microbes growing in the WB, which may easily happen for example in phosphate buffers during longer storage.

## EMBEDDING IN AGAR AND ORIENTATION OF SAMPLES

### Background information

The next steps of sample preparation require serial changing of liquids which in TPs would be exceedingly time consuming, very inconvenient, or from a certain point, even impossible. This is partly because the worm pieces are very small, and also because several of the applied materials are either toxic, very volatile, or even aggressive solvents, which polystyrene cannot withstand. To overcome these difficulties, fixed pieces must be embedded into an appropriate material which keeps the cut parts together and, at the same time, allows the treating agents to properly carry out their action. The usual material for this embedding is either agar, or agarose. They are applied at a concentration which forms a strong enough gel at room temperature for keeping together the pieces against the mechanical forces of sample handling.

We have routinely used 1.5% bacteriological agar liquefied by heating and kept in the water bath of a heating thermostat at 55 °C.

In the stage of agar embedding we included an extra step to achieve the proper orientation of the samples for later longitudinal sectioning while using the most popular flat molds for resin embedding (Sigmond et al. 2008).

### Materials and equipment

- General purpose microbiological agar (e.g., the one used for the *C. elegans* plates)
- 5 cm polystyrene PD for animal tissue culture purposes (TC quality), its bottom is hydrophilic and the agar can be spread more thinly and easily on it (a wide range of companies offer such dishes, for example: Corning, Costar, Greiner, Falcon, Millipore, Nunclon)
- 55 °C water bath
- Thin strips of any type of common, general purpose cellulose filter paper
- Dissecting stereo microscope
- Hypodermic needle of approximately 12–14 gauge
- Small (~10 mL) flat bottomed, clear, glass injection vials (so called headspace vials, ~22 mm outer diameter, ~40 mm height) with wide opening (see for example J.G. Finneran Associates, Inc., 310020-2346) which can be tightly stoppered
- Organic solvent (ethanol and propylene oxide)-resistant test tube caps (see for example SKS Science, T401-4S)
- Graphite pencil
- Adhesive white paper (either tape or sheet, cut into appropriate size)
- Adjustable pipettes
- Hair device
- Razor blade

### Procedure

1. Prepare appropriate amount of 1.5% agar solution in DW and keep it in the 55 °C water bath.
2. Pipette out 1.5 mL warm agar and spread evenly on the bottom of a 5 cm PD by swirling movement until it becomes a firm thin layer.
3. Divide the PD into four quarters by drawing two perpendicular lines on the outside of the bottom, to make areas for four samples. Write the sample number in each quadrant, taking care to leave the central area open.
4. Under the dissecting microscope pipette out the sample pieces from the TP into the appropriate quadrant, close to the center, with 1 µL WB. Take care not to lose any piece.
5. Group the pieces to the edge of the drop by the hair.
6. Remove most of the liquid with a thin strip of filter paper, and group the pieces with the hair device close together so that they touch one another. Remove the rest of the liquid by filter paper again.
7. From a PT, which is trimmed to widen the orifice, let a drop of approximately 15 µL agar fall on the group of samples. Take care that the center of the drop hits the center of the sample group, otherwise the pieces can move away from one another.
8. It is very useful to make a photograph of each sample at this stage.
9. Cut out the sample in a small cube of agar with a razor blade [see also (Sigmond et al. 2008)].
10. Lift the small agar cube, for example by using a hypodermic needle (push the needle under it with the sharp edge oriented downwards) and place it on a free agar area in the same quadrant.
11. Turn the cube by 90° to make the plane of samples perpendicular to the fresh agar surface.
12. Cover the turned agar cube with the samples inside it by adding a new drop of agar.
13. Cut out a block of approximately 2 × 4 mm flat prism with the sample close to one of the edges [see also (Sigmond et al. 2008)].
14. Prepare the 10 mL vials containing ~1–2 mL WB, with an adhesive paper on their side and the sample number written on them with a graphite pencil. Graphite is resistant to organic solvents, which otherwise might remove the sample number if by accident they flow down on the side during later steps of the procedure.
15. Transfer the agar prisms (blocks) into the numbered 8 mL vials with WB, stopper and put them in a refrigerator.

## POSTFIXATION, DEHYDRATION, INFILTRATION, AND POLYMERIZATION

### Background information

As the worm pieces are inside the agar prisms, we can now handle our samples similarly to ordinary tissue blocks. The procedure is carried out by sequential changing of solutions over the agar prisms inside the glass vials. Various solutions for postfixation (staining) dehydration and infiltration are sucked off and the new ones are added. During the process, the small glass vials are put into a slow movement (2 r/min) rotation drum. The whole procedure up to the polymerization must be performed in a ventilated cabinet with good air suction. It is also advisable to use gloves during these processes.

To make thin enough sections from the samples is a prerequisite for utilizing the high resolution of the electron microscope. Sections of approximately 60–90 nm thickness must be prepared to achieve our goal. This requirement is fulfilled by embedding into plastic polymers (embedding resins) which are highly hydrophobic. Therefore, the samples must be dehydrated before getting into the embedding resin. Dehydration is carried out as a stepwise process where a series of ethanol solutions with increasing concentration up to absolute alcohol is changed sequentially over the samples. Finally, propylene-oxide is applied to chemically remove even traces of water. This is followed by the infiltration with the resin. The infiltrated agar blocks are then transferred into the cavities of flat silicone embedding molds and put into a hot thermostat to polymerize.

### Materials and equipment

- Ventilated cabined with efficient air suction
- 0.5% OsO_4_ solution in cacodylate buffer at pH 7.2 (special care is needed with osmic acid as it is very volatile and harmful and should only be used in a chemical hood with proper eye protection)
- 1% uranyl acetate in DW
- Slow speed rotation drum for ~30 samples adjusted to 45° angle
- Narrow stemmed, bulbed, plastic transfer pipette which is organic solvent-resistant
- Ethanol, propylene oxide
- TAAB embedding resin kit with DMP-30 (TAAB Embedding resin kit TK3 T004)
- Toothpicks
- Multispecimen, flat, silicone embedding molds (TAAB Embedding mould type B E071)
- 60 °C degree thermostat

### Procedure

1. Remove the WB using the transfer pipette and replace it with 1 mL 0.5% OsO_4_ solution for postfixation (staining) of about 1 h. The vials must be tightly stoppered because the osmic acid vapors are very volatile and toxic.
2. Remove the OsO_4_ solution and wash twice with DW (~2 mL, for 10 min in each case).
3. Replace DW with 1% uranyl acetate in DW (~1 mL) for the next postfixation (staining) step, soak for 30 min, and also use the stoppers here.
4. Apply a graded series of 50%, 70%, 96% and absolute ethanol (~2 mL in each case) for initial dehydration, leaving the samples in each solution for 20 min, changing the absolute ethanol twice. Stoppering is not necessary until you begin to use the absolute ethanol.
5. Replace absolute ethanol with propylene oxide and leave it on for 20–30 min.
6. Prepare the embedding resin before the end of dehydration, at an appropriate time. Many types of embedding resins are in use and can work well. We presently use the “TAAB embedding resin kit with DMP-30” with the following proportion of the components for 10 mL resin: TAAB resin 5 mL, DDSA 3.8 mL, and MNA 1.2 mL.
7. Mix the components thoroughly to have a homogenous solution, add 0.2 mL DMP and mix well again.
8. Dilute the above resin with propylene oxide by adding one part propylene oxide to three parts of resin.
9. Replace the propylene oxide with the diluted resin of ~1.5 mL for initial infiltration, and leave the samples in the rotating drum for 4–5 h. Use stoppers!
10. Prepare resin with the same proportion of components as above before the end of the initial infiltration, at an appropriate time, and replace the propylene oxide-resin solution with ~1.5 mL of this freshly prepared pure resin.
11. Leave the vials containing the samples in the resin in the rolling rotation drum overnight. We cover the whole bunch of vials with a sheet of aluminum foil and do not use stoppers to facilitate the escape of traces of propylene oxide.
12. The next morning, transfer the infiltrated agar blocks into the cavities of the embedding mold.
13. Prior to placing the blocks into the cavity of the mold, take some of the resin from the vial with a transfer pipette and fill the cavity to more than half with it.
14. Fish out the agar block with a toothpick and place it into the cavity containing the resin that has been put in it beforehand.
15. Fill the cavity by adding more resin to make the top level with the plane of the mold, or slightly convex.
16. Check the position of the blocks under a dissecting microscope when all the samples are in the cavities, and arrange the plane of the sample pieces parallel with the upper side of the cavity (e.g., with a pin). This is important to ensure the precision of the later longitudinal sectioning.
17. Place the molds into a 60 °C thermostat for polymerization for 2–3 days.

## FURTHER TREATMENT OF THE SAMPLES

The remaining steps of sample handling are not special for *C. elegans* and are almost exactly the same as those of ordinary tissue pieces. The plastic blocks with the embedded samples must be trimmed to allow them being accessed for later sectioning. We do this by hand with a razor blade under a dissecting microscope (Sigmond et al. 2008). Approaching must be done very carefully, because the sample is so thin that it can be easily cut off. We have to get as close as possible without actually cutting into the worm pieces. The approaching is finished in the ultramicrotome, when signs of cutting the uppermost regions of a piece appear in a section.

The quality of embedding can be checked partly, when manual trimming is being done, and partly when the first sections have been made in the ultramicrotome. Sometimes samples may appear softer than optimal, then putting them back at this stage into the 60 °C thermostat for a couple of days can improve their quality.

Sections are collected to cover five grids and stained in alkaline lead citrate solution prepared according to Reynolds (Reynolds 1963). Sometimes sections may appear to be somewhat fragile and small cracks can appear in them, especially next to the cuticule, which can only be seen in the electron microscope. In these cases, carbon coating in a vacuum evaporator can substantially stabilize the sections and make them resistent to the electron beam.

## Abbreviations

TEM: Transmission electron microscopy
TP: Terasaki plate
WB: Washing buffer
DW: Distilled water
PD: Petri-dish
FB: Fixative buffer
PT: Pipette tip
